# Feasibility and Initial Outcomes of a Group-Based Teletherapy Psychiatric Day Program for Adults With Serious Mental Illness: Open, Nonrandomized Trial in the Context of COVID-19

**DOI:** 10.2196/25542

**Published:** 2021-03-11

**Authors:** Ajeng J Puspitasari, Dagoberto Heredia, Brandon J Coombes, Jennifer R Geske, Melanie T Gentry, Wendy R Moore, Craig N Sawchuk, Kathryn M Schak

**Affiliations:** 1 Department of Quantitative Health Sciences Mayo Clinic Rochester, MN United States

**Keywords:** COVID-19, teletherapy, intensive outpatient, serious mental illness, mental health, therapy, telemedicine, telehealth, feasibility, outcome, behavioral science, pilot, implementation, effective

## Abstract

**Background:**

In the context of the COVID-19 pandemic, many behavioral health services have transitioned to teletherapy to continue delivering care for patients with mental illness. Studies that evaluate the outcome of this rapid teletherapy adoption and implementation are pertinent.

**Objective:**

This single-arm, nonrandomized pilot study aimed to assess the feasibility and initial patient-level outcomes of a psychiatric transitional day program that switched from an in-person group to a video teletherapy group during the COVID-19 pandemic.

**Methods:**

Patients with transdiagnostic conditions who were at risk of psychiatric hospitalization were referred to the Adult Transitions Program (ATP) at a large academic medical center in the United States. ATP was a 3-week intensive outpatient program that implemented group teletherapy guided by cognitive and behavioral principles delivered daily for 3 hours per day. Feasibility was assessed via retention, attendance rate, and rate of securing aftercare appointments prior to ATP discharge. Patients completed standardized patient-reported outcome measures at admission and discharge to assess the effectiveness of the program for improving quality of mental health, depression, anxiety, and suicide risk.

**Results:**

Patients (N=76) started the program between March and August of 2020. Feasibility was established, with 70 of the 76 patients (92%) completing the program and a mean attendance of 14.43 days (SD 1.22); also, 71 patients (95%) scheduled at least one behavioral health aftercare service prior to ATP discharge. All patient-level reported outcomes demonstrated significant improvements in depression (95% CI –3.6 to –6.2; Cohen *d*=0.77; *P*<.001), anxiety (95% CI –3.0 to –4.9; Cohen *d*=0.74; *P*<.001), overall suicide risk (95% CI –0.5 to –0.1; Cohen *d*=0.41; *P*=.02), wish to live (95% CI 0.3 to 1.0; Cohen *d*=0.39; *P*<.001), wish to die (95% CI –0.2 to –1.4; Cohen *d*=0.52; *P*=.01), and overall mental health (95% CI 1.5 to 4.5; Cohen *d*=0.39; *P*<.001) from admission to discharge.

**Conclusions:**

Rapid adoption and implementation of a group-based teletherapy day program for adults at risk of psychiatric hospitalization appeared to be feasible and effective. Patients demonstrated high completion and attendance rates and reported significant improvements in psychosocial outcomes. Larger trials should be conducted to further evaluate the efficacy and effectiveness of the program through randomized controlled trials.

## Introduction

### Background

Despite 50 years of research examining the utility of telehealth technologies, video teleconferencing has a poor record of implementation, with slow, uneven, and fragmented uptake into routine health care operations [1]. The COVID-19 pandemic prompted behavioral health providers to abruptly shift to video teleconferencing to deliver critical mental health services while supporting statewide stay-at-home orders and physical distancing measures. The quick adoption of telehealth was of particular relevance for transitional behavioral health programs, given that during times of crisis, intensive outpatient programs (IOPs) and partial hospitalization programs (PHPs) play a critical role in preventing psychiatric hospitalization and relapse among patients with serious mental illness (SMI) [2]. Although the impact of the COVID-19 pandemic on people with SMI is still being studied, early evidence indicates that these individuals are experiencing worsening psychiatric symptoms in response to the pandemic [3-5]. Transitioning existing group interventions to video teleconferencing in a systematic manner is one way to adapt clinical practice to meet the needs of vulnerable patients during the pandemic.

Video teleconferencing group psychotherapy and support have been shown to be feasible and have produced similar outcomes to in-person care while maintaining high levels of patient satisfaction [6]. Video teleconferencing groups have used a variety of therapeutic interventions, including cognitive behavioral therapy, acceptance and commitment therapy, and mindfulness. Video teleconferencing groups have also been shown to replicate therapeutic group processes such as a sense of cohesion [7]. To date, few studies have examined the feasibility and efficacy of delivering transitional video teleconferencing group programs to adults with SMI who were recently discharged from or are at high risk of psychiatric hospitalization. One randomized controlled trial (RCT) found that patients with schizophrenia who received intensive case management via video teleconferencing had significantly fewer hospitalizations compared to those who received face-to-face case management and telephone calls from nurses [8]. Furthermore, a descriptive study found a 50% decline in the number of readmissions and fewer days spent in the hospital after implementing a video teleconferencing follow-up program for discharged older adults [9].

SMI is defined as “a mental, behavioral, or emotional disorder resulting in serious functional impairment, which substantially interferes with or limits one or more major life activities” [10]. Adults living with an SMI, such as bipolar disorder or recurrent major depression, are at increased risk for substance abuse, homelessness, and death by suicide [10]. SMI with psychiatric instability accounts for disproportionately high numbers of emergency department (ED) visits and hospital admissions [11]. With regard to psychiatric hospitalization, suicide rates have been shown to be approximately 100 times the global rate during the first 3 months after discharge, and patients admitted with suicidal thoughts or behaviors have been reported to have suicide rates near 200 times the global rate [12]. The provision of step-down, transitional treatment may reduce suicide risk, psychiatric readmission, and the financial burden associated with acute psychiatric care [13-15]. Likewise, offsetting hospital admissions during pandemic times reduces potential exposure risk for SMI patients while simultaneously freeing up inpatient resources to manage census volumes. As such, there is a pressing need to rapidly restructure face-to-face transitional behavioral health programs for digital delivery to ensure safe and socially distant access to evidence-based care during the COVID-19 public health emergency.  

Best-practice recommendations for post–psychiatric hospital discharge and prevention of psychiatric hospital admission includes IOP and PHP interventions that implement evidence-based psychotherapeutic treatments such as cognitive behavioral therapy (CBT) [16] or dialectical behavioral therapy (DBT) [17]. Existing studies have demonstrated that patients with transdiagnostic psychiatric conditions who received short-term, intensive outpatient treatment showed clinical improvements and used fewer inpatient psychiatric and emergency medical services [2]. These findings, however, are based on in-person programs, and few studies have examined the feasibility and effectiveness of video teleconferencing delivery of transitional programs for adults with acute psychiatric needs.

### Objectives

The present study evaluated the feasibility and initial effectiveness of the Adult Transitions Program (ATP), a group-based teletherapy IOP for adults with transdiagnostic conditions who are at risk for psychiatric hospitalization. After the COVID-19 pandemic started, ATP rapidly switched from an in-person to a teletherapy format to assure patient and staff safety and to maintain continuity of care. Feasibility was defined in terms of the number of patients who completed the program, average days of attendance, and securing of behavioral health aftercare services prior to ATP discharge. Effectiveness was assessed using standardized patient-reported outcome (PRO) measures of quality of mental health and symptoms of depression, anxiety, and suicidality completed at admission and discharge. It was hypothesized that patients would demonstrate high feasibility of ATP and report significant clinical improvement in psychosocial outcome measures. If supported, the findings could further justify the delivery of ATP through the teletherapy format for patients with acute psychiatric needs.

## Methods

### Procedures

The study protocol was approved by the Mayo Clinic’s institutional review board. This open trial used an observational, retrospective cohort study design. Patients were referred to the program from the ED, inpatient psychiatric hospital units, and outpatient behavioral health services. ATP implemented a rolling admission process, with this study including patients who were admitted into the program between March and August 2020. Eligibility to enter the program was determined by either an in-person or video teleconferencing preprogram evaluation delivered by either licensed professional clinical counselors (LPCCs) or registered nurses (RNs).

If patients were deemed eligible for the program, they were assigned to one of the three tracks (see the Program Description section) by an LPCC who managed the triage and admission process. Prior to the start of the program, patients received a therapy binder delivered via mail or picked up by the patients at the hospital. As part of routine clinical practice, patients completed PRO measures electronically at admission and discharge. Program staff sent these measures via the electronic health record (EHR, ie, EPIC), and the results were automatically incorporated to the patients’ EHRs. An LPCC reviewed the patients’ PRO measure scores at admission and discharge with the patients, and the results were used to guide treatment planning and progress monitoring as part of the program’s effort to implement measurement-based care [18]. A chart review was conducted to obtain the demographics, feasibility, and effectiveness data from the patients’ EHRs.

### Inclusion and Exclusion Criteria

Inclusion criteria were adults (aged 16-65 years) who had transdiagnostic psychiatric conditions (eg, mood disorders, anxiety disorders, psychosis, personality disorders, and substance use); were at risk for psychiatric hospitalization or rehospitalization; and reported having access to a mobile or computer device to connect to the video teleconferencing software (ie, Zoom). Exclusion criteria from the program were cognitive impairment that prevented participation in the group format and higher symptom severity that was more appropriately addressed in a higher level psychiatric inpatient or residential setting. Data from patients who did not provide authorization for their clinical data to be used for research purposes were also excluded from the final analysis.

### Program Description

ATP was developed as a short-term IOP to bridge patients who were recently discharged from, or at risk for, psychiatric hospitalization. The original program started in 2013 and was delivered in-person. Due to the COVID-19 pandemic, the program underwent a rapid transition to video teleconferencing in March 2020. We referred to available guidelines on teletherapy [19-21] as well as to a model to select and use strategies to facilitate successful teletherapy adoption and implementation [22]. The multidisciplinary team consisted of a clinical director/clinical psychologist, medical director/psychiatrist, nurse practitioners/physician assistants (NPs/PAs), LPCCs, occupational therapists, and RNs. Psychotherapy groups were led by LPCCs who attended a weekly consultation meeting facilitated by a doctoral-level clinical psychologist to ensure treatment adherence and fidelity. All disciplines attended daily huddles to discuss safety management and patient progress.

The ATP video teleconferencing format was delivered 5 days per week, 3 hours per day, and it consisted of primarily group-based interventions with rolling admission. The two primary goals of the program were to provide immediate practical skills to manage symptoms and support recovery and to transition patients to the appropriate level of care (eg, outpatient therapy, substance use treatment, an intensive DBT program). As such, we adapted our group psychotherapy content based on several evidence-based CBT principles, which included strategies drawn from behavioral activation (BA) [23], a DBT skills group [24], and process-based therapy [25].

Because the duration of the program was relatively short, we selected strategies from these different modalities and delivered them to patients modularly. We did not make any changes in the curriculum when we transitioned to the video teleconferencing group format. The program consisted of three tracks, with 8 patients in each track at any given time. Track 1 was designed for patients with comorbid psychiatric disorders and addiction who might also struggle with suicidality. Group interventions included a BA group that focused on selecting daily goals and scheduling activities (eg, self-care, pleasurable activities, social activities, and activities to increase mastery and productivity); a recovery group for addiction guided by the DBT model [26]; and a DBT skills group [24]. Of note, because a typical DBT skills group is delivered in 6 months to 1 year, we did not cover the full DBT skills group manual. Instead, we selected several strategies from each of the four DBT skills core modules: mindfulness, emotion regulation, interpersonal effectiveness, and distress tolerance. Specifically, our clinical workgroup selected skills that are most relevant for our patient population (ie, those who are at risk of psychiatric hospitalization) and could be readily used in the short time they were in the program (ie, 3 weeks). For example, instead of going through all interpersonal effectiveness skills, our program focused on DBT assertiveness skills such as DEAR MAN (Describe, Express, Assert, Reinforce, Stay Mindful, Appear Confident, Negotiate) because asking for help and being assertive are commonly occurring barriers for our patients.

Track 2 was designed for patients with transdiagnostic psychiatric conditions who struggled with high suicidality and self-injurious behaviors. Similar to Track 1, this track consisted of a BA group and a DBT skills group; however, instead of a recovery group, an occupational therapy (OT) group was included in programming. Track 3 was primarily designed for patients with anxiety and depression with less acuity than those in Tracks 1 and 2. The treatment modality for this group was mainly guided by a process-based therapy model [25] that included evidence-based CBT principles to target common psychological challenges (eg, cognitive fusion, low motivation to create behavioral change, and avoidance). Strategies from acceptance and commitment therapy [27] and CBT are incorporated into modular topics in this track.

In addition to receiving three group teletherapy sessions per day, patients also received individual sessions throughout their enrollment in the program. Each patient was assigned a primary LPCC who was responsible for developing the treatment plan, providing individual interventions when needed, monitoring progress, and preparing for aftercare. On average, patients met with an LPCC at three time points: admission, midpoint check-in, and discharge. Patients also met with an NP/PA at least once during admission to discuss medications. Those who were assigned to Track 2 received OT evaluation at admission. If needed, additional individual sessions could be arranged while patients were in the program. Almost all individual sessions were delivered via video teleconference, unless an in-person session was deemed to be more appropriate.

As many patients struggled with suicidal behaviors, strategies from the Collaborative Assessment to Manage Suicidality [28] were implemented in individual sessions, as needed. All patients who were accepted to the program completed the Suicide Status Form (SSF) during the preprogram evaluation. If elevated suicide ideation and intent were observed, patients met with an LPCC to develop a safety and stabilization plan and determine strategies to use to cope with suicidal behaviors (eg, distress tolerance skills, asking for social support, distraction). We provided information to a crisis line or recommended patients to go to the nearest ED if their suicide ideation and intent could not be managed independently.

### Video Teleconference Support

Patients who were accepted into the program received assistance from ATP staff and information technology support staff to prepare for the first group video teleconferencing. Patients were asked to set up the required Health Insurance Portability and Accountability Act (HIPAA)–approved encrypted software, Zoom, on their device. Patients were able to use their smartphone, laptop, or computer. Assistance included a test call to teach patients how to use the video teleconferencing platform and to ensure adequate connectivity.

Prior to the start of the program, patients received the link to the group video conference room. When patients logged into Zoom, they were automatically placed in the waiting room to assure that only those who were enrolled in the program were accepted to the chat room by the facilitators. Each psychotherapy group was led by two LPCCs; one functioned as the primary facilitator who led the presentation and group discussion, while the other assisted patients with any technological issues. The program consisted of open group admission, and when a new patient started the program, the facilitator went through the group norms, which included but were not limited to the importance of attending group video teleconferences in a private area and turning on their camera to assure confidentiality.

Several features that were routinely used over Zoom were the chat feature, whiteboard, shared screen for slide presentations and videos, and the waiting room in which we placed patients during breaks between group psychotherapy sessions. It was also feasible to conduct psychotherapy experiential exercises via video teleconferencing, such as performing guided group mindfulness exercises, completing psychotherapy forms, and watching psychotherapy skills videos.

### Measures

All measures were completed via the EHR through the patient portal. Sociodemographic data included biological sex, age, ethnicity, gender identity, sexual orientation, marital status, employment status, and financial resource strain. Program feasibility was defined as the rate of patients who completed the program. Our benchmark for feasibility was that 75% of patients would complete the three-week program and attend at least 8 out of 15 days. Because ATP was developed to assure that patients had an appropriate continuum of care, another benchmark for feasibility was to assure that patients had at least one behavioral health service appointment after ATP discharge.

Patients completed four primary outcome measures at admission and discharge to assess program effectiveness. Overall quality of mental health was measured using the Patient-Reported Outcomes Measurement Information System (PROMIS) Global-10 [29] to assess overall physical and mental health. This 10-item scale has been shown to be reliable, precise, and comparable to legacy instruments. In this study, we report only the overall mental health *t* scores ranging from 0 to 100 with the following cut points of poor, fair, good, very good, and excellent [30]. Depressive symptoms were measured using the Patient Health Questionnaire-9 (PHQ-9) [31], a 9-item scale with a total score range from 0-27 wherein higher scores are suggestive of greater depressive symptoms; scores of 5, 10, 15, and 20 represent mild, moderate, moderately severe, and severe depression, respectively. Anxiety symptoms were measured using the Generalized Anxiety Scale-7 (GAD-7) [32], a 7-item scale with a total score range from 0 to 21 wherein higher scores are suggestive of greater anxiety symptoms; scores of 5, 10, and 15 represent mild, moderate, and severe anxiety, respectively. Finally, suicide risk, wish to live, and wish to die were measured using the SSF [33]. Suicide risk was reported on a scale of 1 (low) to 5 (high). Wish to live and wish to die were reported on a scale of 0 (low) to 8 (high).

### Statistical Analyses

Feasibility was measured by calculating the percentage of patients who completed the program. Average days attended was also calculated as another metric for feasibility. Finally, as a benchmark for feasibility, we calculated the percentage of patients who secured at least one behavioral health service appointment prior to ATP discharge. Paired *t* tests were used to examine changes from admission to discharge in the psychosocial outcomes of quality mental health, depression, anxiety, and suicidality. These analyses included only patients who completed both the admission and discharge measures (n=60). *P* values <.05 were considered significant. A linear model was used to assess differences in symptom improvement for patients in the three different tracks by regressing the group indicator onto the change in symptom score from baseline to discharge and adjusting for baseline score. Data were analyzed using SAS, version 9.4 (SAS Software).

## Results

### Baseline Characteristics

Patients had a mean age of 36.6 years (SD 13.4), ranging from 18 to 73 years in age. The majority of the 76 patients were female (n=65, 86%) and White (n=69, 91%), married (n=22, 29%) or single (n=44, 57.9%), cisgender (n=73; 96%), heterosexual (n=52; 68%), and employed (n=46, 61%). The 76 patients had the following psychiatric diagnoses as a primary presenting problem: major depressive disorder (n=52, 68%), bipolar disorder (n=6, 8%), anxiety disorder (n=22, 29%), personality disorder (n=13, 17%), substance use disorder (n=6, 8%), and schizophrenia (n=2, 3%). The majority of patients had comorbid psychiatric diagnoses (n=41, 54%). The full baseline characteristics of the sample are reported in Table 1.

**Table 1:** Baseline characteristics of the study sample (N=76).

Characteristic			Value
Age (years), mean (SD)			36.55 (13.43)
**Sex, n (%)**			
	Female	65 (86)	
	Male	11 (15)	
**Gender, n (%)**			
	Female	63 (83)	
	Male	10 (13)	
	Transgender female	2 (3)	
	Transgender male	1 (1)	
**Race, n (%)**			
	White	68 (90)	
	Other	5 (7)	
	African American	2 (3)	
	Chose not to disclose	1 (1)	
**Ethnicity, n (%)**			
	Hispanic or Latino	4 (5)	
	Non-Hispanic or Latino	69 (91)	
	Puerto Rican	1 (1)	
	Chose not to disclose	1 (1)	
	Unknown	1 (1)	
**Marital status, n (%)**			
	Single	44 (58)	
	Married	22 (29)	
	Separated	4 (5)	
	Widowed	1 (1)	
	Divorced	5 (7)	
**Employment, n (%)**			
	Currently employed	46 (61)	
	Not employed	27 (36)	
	Disabled	3 (4)	
**Financial resource strain, n (%)**			
	Not hard at all	23 (30)	
	Not very hard	14 (18)	
	Somewhat hard	21 (28)	
	Hard	5 (7)	
	Very hard	9 (12)	
	Patient refused to answer	1 (1)	
	Not on file	3 (4)	
**Sexual orientation, n (%)**			
	Lesbian or gay	3 (4)	
	Heterosexual	52 (68)	
	Bisexual	4 (5)	
	Other	1 (1)	
	Don’t know	14 (18)	
	Chose not to disclose	2 (3)	
**Presenting problems, n (%)**			
	Major depressive disorder	52 (68)	
	Bipolar disorder	6 (8)	
	Anxiety disorder	22 (29)	
	Personality disorder	13 (17)	
	Substance use disorder	6 (8)	
	Schizophrenia	2 (3)	
**Comorbidity, n (%)**			
	With one diagnosis	35 (46)	
	With comorbid diagnoses	41 (54)	
**Track, n (%)**			
	Dialectical behavioral therapy AM	26 (34)	
	Dialectical behavioral therapy PM	29 (38)	
	Cognitive behavioral therapy AM	20 (26)	
**Source of referral, n (%)**			
	Inpatient	26 (34)	
	Emergency department	3 (4)	
	Primary care	23 (30)	
	Other outpatient	19 (25)	
	Other programs	5 (7)	
Days completed, mean (SD)			14.43 (1.22)
Medication management appointment scheduled, n (%)			67 (88)
Group psychotherapy appointment scheduled, n (%)			10 (13)
Case management scheduled, n (%)			6 (8)
Residential treatment scheduled, n (%)			1 (1)
Substance use disorder treatment scheduled, n (%)			1 (1)
Referred for therapy, n (%)			3 (4)
Referred for group therapy, n (%)			13 (17)
Referred for substance use disorder treatment, n (%)			1 (1)
Individual psychotherapy or outpatient therapy, n (%)			59 (78)
**Program absences (days), n (%)**			
	None	49 (65)	
	1-3	18 (24)	
	4-7	3 (4)	
	Noncompleters	6 (8)	

### Program Feasibility

**Figure 1:**
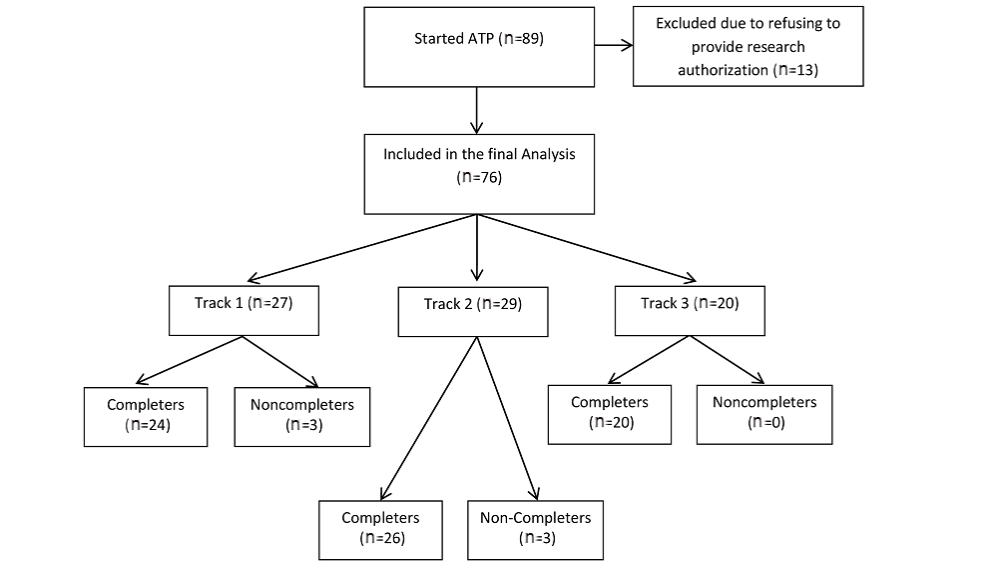
Participant enrollment and completion of the video teleconferencing ATP. ATP: Adult Transitions Program.

Figure 1 delineates the flow of patients in this study. From the initial 89 patients who started the program, 13 were excluded because they declined to have their clinical data used as part of research. Thus, the completion rate of the program was 70/76 (92%). Completion was defined as patients who attended at least 50% of the sessions [34]. The number of attended sessions ranged from 8 to 15, and patients completed an average of 14.38 days (SD 1.42). In terms of aftercare post–ATP discharge, 93% (71/76) of patients scheduled at least one behavioral health service appointment. Specifically, 74% (56/76) received outpatient psychotherapy and medication management, 13% (10/76) received at least outpatient psychotherapy or medication management and group psychotherapy, 8% (6/76) only had medication management, and 3% (2/76) only received outpatient psychotherapy.

### Program Effectiveness

Improvement was observed for all PRO measures (Table 2). Depression scores improved from a mean of 14.4 (SD 6.5) at admission to 9.6 (SD 6.0) at discharge (PHQ-9 change, 95% CI –3.6 to –6.2; Cohen *d*=0.77; *P*<.001). Likewise, patients reported improvements in self-reported anxiety, from 11.8 to 7.7 (GAD-7 change, 95% CI –3.0 to –4.9; Cohen *d*=0.74; *P*<.001). In terms of suicidality, patients reported significant improvements in overall suicide risk (SSF change, 95% CI –0.5 to 0.1; Cohen *d*=0.41; *P*=.02), wish to live (SSF change, 95% CI 0.3 to 1.0; Cohen *d*=0.39; *P*<.001), and wish to die (SSF change 95% CI –0.2 to –1.4; Cohen *d*=0.52; *P*=.01). Finally, patients also reported significant improvement in their overall mental health, from 40.2 at admission to 42.0 at discharge risk (PROMIS Global 10 change, 95% CI 1.5 to 4.5; Cohen *d*=0.39; *P*<.001). Additionally, we conducted a stratified analysis to compare changes in PRO measure outcomes between patients in the three different tracks. There were no statistically significant differences in the outcome measure changes across the tracks.

**Table 2:** Changes in standardized patient reported outcome measures from admission to discharge (N=76).

Measure	Responses (n)	Mean score (SD)		*P* value
		Admission	Discharge	
Patient Health Questionnaire-9	60	14.40 (6.47)	9.58 (6.04)	<.001
Generalized Anxiety Disorder-7	60	11.80 (5.57)	7.67 (5.52)	<.001
Alcohol Use Disorder Identification Test (AUDIT)	55	2.80 (3.84)	2.47 (3.60)	.18
Wish to live	58	6.26 (1.81)	6.95 (1.69)	<.001
Wish to die	58	1.67 (2.15)	0.74 (1.29)	.01
Risk of suicide	58	1.53 (0.82)	1.24 (0.58)	.02
PROMIS^a^ Global 10	37	40.19 (4.43)	42.04 (5.02)	<.001

^a^PROMIS: Patient-Reported Outcomes Measurement Information System.

## Discussion

### Key Findings

The aim of this open, nonrandomized, retrospective trial was to assess the feasibility and initial effectiveness of ATP, an IOP delivered via video teleconferencing for adults with SMI who were at risk for psychiatric hospitalization. The rapid switch of the program from in-person to video teleconferencing was a response to the start of the COVID-19 pandemic during the initial months of 2020. Below, we discuss our key findings along with limitations and future directions.

The feasibility of offering ATP via video teleconferencing was evaluated primarily in terms of completion rate (ie, attending at least 8 out of 15 program days), average days attended, and securing at least one behavioral health service appointment prior to ATP discharge. Based on the 76 patients who were included in the final analysis, 70 patients (92%) completed the program. This completion rate was higher than typical completion rates for psychiatric IOP or PHP programs, which are <60% [35,36]. Furthermore, the average number of days completed by patients was 14.43 (SD 1.22), which indicated that the majority of patients only missed approximately 1 day in the three-week program. Because continuation of care has been shown to be an important predictor of future relapse and functional recovery [37], we selected securing aftercare appointments prior to ATP discharge as the last benchmark of program feasibility. Our chart review indicated that the vast majority of patients scheduled at least one behavioral health service appointment before discharge. The most frequently scheduled aftercare appointments included either medication management or psychotherapy. Thus, these three metrics indicated that it was feasible to deliver ATP via video teleconferencing, as demonstrated by the high completion rate, good attendance, and successful aftercare transition. However, we could not conclude how these feasibility outcomes compared to in-person iterations of ATP.

With regard to effectiveness, overall quality of mental health, symptoms of depression, anxiety, wish to live, wish to die, and reported suicide risk improved from admission to discharge. The effect sizes for the outcome measures ranged from small to moderate effect sizes. Because patients were assigned to one of the three tracks, we conducted a stratified analysis to assess potential differences in symptom changes by track. The results indicated no significant differences in the changes of the standardized PRO measure scores between the three different tracks, suggesting that both the shared and differing content across the tracks were similarly effective in reducing distress and improving quality of life.

### Limitations and Future Directions

This study has several limitations that should be discussed. First, the lack of a control condition limits our ability to firmly conclude that the positive feasibility outcomes and changes in PRO measures were solely due to the program interventions and/or delivery mode. To assess the efficacy of ATP delivered via video teleconferencing format, an RCT is warranted. A larger RCT should include comparison groups, such as ATP delivered via an in-person format, or a wait-list control condition. Second, a lack of follow-up data limited our understanding of the long-term impact of ATP delivered via video teleconferencing to sustain improvements in overall mental health, depression, anxiety, and suicidal behaviors. Long-term follow-up would also be important to assess whether patients who completed ATP adhered to the aftercare recommendations and reduced the rate of psychiatric hospitalization post–ATP discharge. Third, although the effectiveness analysis showed encouraging results, this was based on a completer analysis. Furthermore, although it is not possible to determine if the missing data affected the results positively or negatively, missing data appeared to occur at random, and individuals with missing data were no different as a group than those without missing data in terms of demographic variables such as age, gender, and other baseline variables. Future studies should include a more rigorous protocol to treat missing data, such as follow-up with patients who prematurely dropped out or did not complete the PRO measures at different time points.

### Conclusion

This pilot investigation demonstrated the feasibility and initial effectiveness of ATP, a program that was rapidly switched to a video teleconferencing format during the COVID-19 pandemic. The majority of patients completed the program and demonstrated high attendance rates. It was feasible to secure aftercare behavioral health appointments prior to program discharge to ensure continuation of care. Patients also reported improvements in self-reported measures of mental health, psychiatric symptoms, and quality of life. This finding is encouraging because a rapid transition to video teleconferencing during a world pandemic may reduce the mental health crisis that has been predicted to soon follow. The delivery of group-based IOP via video teleconferencing that implemented evidence-based cognitive and behavioral principles for patients with transdiagnostic psychiatric conditions is a promising strategy to improve access to behavioral health services during and after the COVID-19 pandemic. Because this is a pilot trial, the results and conclusion should be taken carefully. Larger trials should be conducted to further test the significance of this program model, its cost-effectiveness, and its efficacy to reduce the public health burden of mental illness, particularly in the context of a world pandemic such as COVID-19.

## Abbreviations

ACT: acceptance and commitment therapy
ATP: Adult Transitions Program
BA: behavioral activation
CBT: cognitive behavioral therapy
DBT: dialectical behavior therapy
DEAR MAN: Describe, Express, Assert, Reinforce, Stay Mindful, Appear Confident, Negotiate
ED: emergency department
EHR: electronic health record
GAD-7: Generalized Anxiety Disorder Scale-7
HIPAA: Health Insurance Portability and Accountability Act
IOP: intensive outpatient program
LPCC: licensed professional clinical counselor
NP: nurse practitioner
OT: occupational therapy
PA: physician assistant
PHP: partial hospitalization program
PHQ-9: Patient Health Questionnaire-9
PRO: patient-reported outcome
PROMIS: Patient-Reported Outcomes Measurement Information System
RN: registered nurse
RTC: randomized controlled trial
SMI: serious mental illness
SSF: Suicide Status Form
SUD: substance use disorder
